# Comparison of Pain Scores and Medication Usage Between Three Pain Control Strategies for Pediatric Anterior Cruciate Ligament Surgery

**DOI:** 10.7759/cureus.5498

**Published:** 2019-08-27

**Authors:** Lisgelia Santana, John F Lovejoy, Gary Kiebzak, Jason Day, Alfred Atanda Jr., David Mandel

**Affiliations:** 1 Anesthesiology and Pain Management, Nemours Children's Hospital, Orlando, USA; 2 Orthopedics and Sports Medicine, Nemours Children's Hospital, Orlando, USA; 3 Orthopedic Surgery, Nemours Children's Hospital, Orlando, USA; 4 UCF College of Medicine, Orlando, USA; 5 Orthopedic Surgery, Nemours/Alfred I. duPont Hospital for Children, Wilmington, USA; 6 Orthopedic Surgery, Nemours Children's Specialty Care, Jacksonville, USA

**Keywords:** anterior cruciate ligament, opioid, pediatric pain, pediatric surgery, postoperative pain, visual analogue scale, regional anesthesia

## Abstract

Introduction

Assessment and management of postoperative pain in the pediatric population after anterior cruciate ligament (ACL) surgery can be challenging; the optimal approach to pain control remains controversial. Recent studies show that use of intraoperative nerve blocks may reduce the need for opioids to control pain in the postoperative period. However, it is unclear which block type is most beneficial in the pediatric outpatient setting. This study compared effectiveness of pain control among three different pain management strategies.

Methods

We retrospectively reviewed charts of patients aged 12-17 years who received an elective ACL reconstruction between 2013 and 2017. The three groups were femoral nerve block, combined femoral and sciatic block, and intraarticular injection of bupivacaine (n = 50 per group). The primary variable was postoperative pain scores (visual analog scale 1-10) in the postanesthesia care unit (PACU).

Results

Less than 50% of patients in the combined nerve block group had opioids intraoperatively or in the PACU compared with nearly 100% of patients in the other two groups (p < 0.0001). Also, for patients receiving opioids, the total intraoperative morphine equivalents and PACU pain scores (all patients) were significantly less in the combined block group (p < 0.001). For patients receiving opioids in the PACU, the total morphine equivalents were significantly higher in the intraarticular injection group compared with the nerve block groups (p < 0.0001).

Conclusion

Patients in the combined femoral and sciatic nerve block group had significantly better pain scores in the PACU with less cumulative morphine equivalent consumption compared with the femoral nerve block group and the intraarticular injection group.

## Introduction

Increased use of regional anesthesia in infants, children, and adolescents has significantly improved the scope of pediatric pain management. Performance of regional anesthesia can be safely and effectively applied to pediatric patients, and these techniques are becoming increasingly popular [1-3]. As a result, regional anesthesia is becoming accepted as an integral component of postoperative pain relief in pediatric patients [1].

Prior to availability of ultrasound imaging for regional anesthesia, many providers were reluctant to perform peripheral nerve blockade (PNB) in the pediatric population. Regional techniques utilizing landmarks or nerve-stimulation methods were typically avoided in children due to the difficulty in targeting neural structures that are often in close proximity to other critical structures [4]. These patients frequently needed general anesthesia or sedation, which can potentially mask complications as well.

Following the introduction of ultrasound-guided nerve localization, regional anesthetic techniques gained popularity as a method to improve pain management among children undergoing orthopedic procedures. A study using large prospective databases has demonstrated the ability to safely perform regional anesthesia in children with minimal risk of neurological damage using sedation or general anesthesia [5]. In addition, the use of ultrasound guidance and its incorporation into the practice of regional anesthesia has dramatically improved routine pediatric perioperative care. The use of regional blockade affords the anesthesiologist the ability to reduce, if not eliminate, the need for parenteral opioids along with opioid-induced nausea and vomiting, a common cause of discharge delay after ambulatory surgery [6]. The reduction of opioids during the initial recovery phase can also potentially contribute to decreased opioid use after discharge in this young population, decreasing risks of diversion and addiction.

Advanced pain management techniques are associated with a unique set of risks and benefits. For example, although a femoral and sciatic nerve block would provide complete postoperative analgesia after a knee arthroscopy for anterior cruciate ligament (ACL) repair, these techniques may be associated with perioperative nerve injury, prolonged dysesthesias, motor weakness, or bleeding and infectious complications [7]. In contrast, the use of intraarticular infiltration may minimize concerns related to nerve injury, dysesthesias, and motor weakness but at the potential expense of inferior analgesia when compared with PNB.

Clinical trials comparing these analgesic modalities with one another are lacking, resulting in a significant knowledge gap and inability to determine which pain management technique is best suited for patients undergoing knee arthroscopy and ACL repair. Therefore, the primary goal of our study was to compare pain control and opioid usage between a combination of PNBs (femoral nerve block versus femoral and sciatic nerve block) versus intraarticular injections of bupivacaine-based local anesthetic.

We hypothesized that pediatric patients undergoing knee arthroscopy for ACL repair will have lower postoperative pain, lower consumption of opioid medications, and faster recovery and hospital discharge with PNB compared with intraarticular injections using bupivacaine in the immediate postoperative period. We also hypothesized that there would be no significant difference in the immediate postoperative outcome between use of a femoral nerve block with the combined femoral and sciatic nerve block combination.

## Materials and methods

We performed a multisite retrospective chart review consisting of patients with American Society of Anesthesiology (ASA) I and II with three different pain management strategies during an elective unilateral ACL reconstruction (femoral nerve block, combined femoral and sciatic block, or intraarticular injection of bupivacaine) between 2013 and 2017. This study was approved by our Institutional Review Board. Patients aged 12 to 17 years who had elective ACL reconstruction were included in this study. There were 50 patients consecutively chosen from the search list per group. The power analysis was based on the differences between femoral nerve block plus sciatic nerve block vs. intraarticular injection, as the literature suggests these may be the groups with the best and worst pain scores (i.e., the extremes of the study groups). A difference of 2 on the pain scale is believed to be clinically significant. Postanesthesia care unit (PACU) pain scores have not been tabulated in previous studies, so we have no historical data to use to estimate the degree of variability (relevant literature suggests standard deviation [SD] equals 2 or 3). To detect a 2-point difference at 90% power, assuming SD equals 2, alpha equals 0.05 (two-tailed), we would need about 25 patients per group. But if we see that the SD is closer to 3 after the first 10 patients, then about 50 patients per group will be required. The primary variable collected was postoperative pain score (visual analog scale [VAS] 1-10, no pain to worst possible pain) every 15 minutes until the patient was discharged home. The pain scores were recorded in the PACU by a single nurse on a one-to-one care basis after the patient awoke from general anesthesia. This score was plotted on a flowsheet in the electronic medical record (Epic Systems Corporation, Verona, WI). Secondary variables collected were demographic data, time in PACU, and intraoperative/postoperative analgesic use.

All patients received the same protocol with intravenous induction with propofol and maintenance of general anesthesia with sevoflurane and laryngeal mask airway placement before the block or intraarticular injection. Blocks were given using ultrasound (SonoSite 180 Plus portable ultrasound unit [FUJIFILM SonoSite, Inc., Bothell, WA]) before the incision. After aseptic preparation of the puncture site and ultrasound probe, the nerve blocks were performed using an insulated 22-gauge needle with facette tip and injection line (PajunkTM, Geisingen, Germany). All blocks were performed with the in-plane technique. The needle was positioned close to the target nerves under ultrasound visualization. The local anesthetic ropivacaine (20 ml, 0.2%) was injected under real-time ultrasound control until the nerve was surrounded. An observer injected the local anesthetic and recorded the injected amount. All patients in the intraarticular injection group received bupivacaine (20 ml, 0.25%) after surgery was completed. At the end of the surgery, the patient was taken to the recovery room and assessment of pain was done every 15 minutes using the VAS until discharge.

Statistical analyses

Descriptive statistics (mean ± standard deviation) were calculated. Results for the three groups were compared using the Kruskal-Wallis nonparametric analysis of variance (ANOVA) test (standard ANOVA could not be used because data were not normally distributed) followed by Dunn’s multiple comparisons test (which compared each group with every other). Percentages were compared using the chi-squared test for trend.

## Results

There was no statistically significant difference in mean age of patients in the three groups: 15.6 ± 1.3, 15.1 ± 1.4, and 15.1 ± 1.4 years for femoral nerve block patients, combined block, and intraarticular injection, respectively. Likewise, there was no difference in body mass index: 26.7 ± 6.4, 24.8 ± 6.0, and 24.8 ± 5.3. The ratio of boys to girls was similar in the three groups, with boys comprising 54%, 40%, and 46% in femoral nerve block patients, combined block, and intraarticular injection, respectively. However, there was a difference in race profiles among the three groups (a manifestation of site location); for the femoral nerve block group, only 28% were Caucasian, compared with 58% and 56% in the combined block group and intraarticular injection group, respectively (p = 0.002).

Table 1 summarizes the use of pain medications and PACU pain scores. Less than 50% of patients in the combined nerve block group had opioids intraoperatively or in the PACU compared with nearly 100% of patients in the other two groups (p < 0.0001). Also, for patients receiving opioids, the total intraoperative morphine equivalents and PACU pain scores (all patients) were significantly less in the combined block group (p < 0.001). For patients receiving opioids in the PACU, the total morphine equivalents were significantly higher in the intraarticular injection group compared with the nerve block groups (p < 0.0001). Total time in the PACU was 25% longer for patients in the combined block group (p < 0.0001).

**Table 1:** Comparison of Three Pain Management Strategies for Anterior Cruciate Ligament Surgery Data are mean ± standard deviation. For each group, n = 50 except as noted (n). Notes: Means for the three groups were compared using the Kruskal-Wallis nonparametric analysis of variance test, followed by Dunn’s multiple comparison test. *The value was significantly different from the others. **Mean calculated for patients who actually received meds, notfor *all* patients, thus sample size is <50. Percentages were compared using the chi-squared test for trend. PACU: Postanesthesia care unit; VAS: Visual analog scale.

Study Group				
Variable	Femoral Nerve Block	Femoral Plus Sciatic Block	Intraarticular Injection	P
Percent getting intraoperative opioid	100	48*	98	<0.0001
Percent getting opioid in PACU	92	28*	96	<0.0001
Intraoperative morphine equivalents, mg**	13.9 ± 5.5 (50)	7.7 ± 8.2 (24)*	13.7 ± 3.8 (49)	<0.0001
PACU morphine equivalents, mg**	5.0 ± 3.6 (46)	4.7 ± 2.6 (14)	10.1 ± 3.9 (48)*	<0.0001
Total PACU time, min	116 ± 44	144 ± 47*	115 ± 56	<0.0001
Highest PACU VAS pain score	6.8 ± 2.2	2.5 ± 3.2*	5.6 ± 2.5	<0.0001
Mean PACU VAS pain score	4.5 ± 2.0	1.7 ± 2.4*	4.1 ± 1.8	<0.0001
Last PACU VAS pain score	2.7 ± 2.0	1.5 ± 2.0*	2.8 ± 1.8	0.0013
Number of PACU scores recorded	4.0 ± 1.7	4.0 ± 1.5	3.0 ± 1.8*	0.0015

## Discussion

The practice of pediatric regional anesthesia has made tremendous strides in recent years. Advances in ultrasonography and precise dosing regimens have facilitated widespread applications of regional anesthetic techniques in infants, children, and adolescents. Prospective studies indicate that these techniques can be safely performed and positively impact the outcomes of pediatric patients undergoing painful procedures [8,9]. Safe dosing guidelines and age-related trends in local anesthetic pharmacokinetics and pharmacodynamics have been characterized and have facilitated expansion of pediatric regional anesthesia practice.

A fundamental knowledge of the anatomical structure of the knee and associated neural innervation is critical to understand the utility and limitations of specific nerve blocks in managing postsurgical pain. Innervation of the knee comes from nerves arising from the lumbar plexus and the sciatic nerve. Femoral nerve block is an effective and safe method of providing postoperative analgesia to children and adolescents undergoing orthopedic knee surgery [10]. In many pediatric hospitals, femoral nerve block is now routinely used for arthroscopic knee surgery. However, other practices include sciatic nerve block (in combination with the femoral nerve block) for a more complete coverage of the knee pain or less invasive techniques, such as intraarticular injections, at the end of the procedure. Other practices include the use of nerve catheters and a pump for the patient to go home and have prolonged pain control for a few days after surgery. To our knowledge, no study has compared these techniques in the pediatric population with regard to the effect on immediate postoperative pain and use of narcotics for pain management.

In our study, the femoral and sciatic nerve block group had statistically significant better pain scores with less cumulative morphine equivalent consumption use compared with the intraarticular injection group and the femoral nerve block group both intraoperatively and in the PACU. However, despite needing less pain medication in the PACU, the double PNB group had a longer stay in recovery. One possible theory explaining this finding is that a combined femoral plus sciatic block is a denser block leaving the patient weaker and insensate longer and thus making it more difficult to meet discharge requirements. That is, the patients with combined blocks likely required more time to perform transfers and learn use of crutches, explaining the longer time in recovery. The ability to provide site-specific analgesia coupled with the ability to reduce opioid requirements produces patients who are comfortable and symptom-free in the recovery room. Moreover, decreasing the time to meet discharge criteria after regional anesthesia likely means that these patients will be more alert during the recovery phase, as decreasing opioid administration diminishes opioid-induced side effects such as drowsiness and somnolence.

New techniques in regional anesthesia are emerging frequently. During the last couple of years, adductor canal blocks have been taking over femoral nerve blocks in some practices. By targeting the distal branches of the femoral nerve in the mid-thigh, the adductor canal block can preserve quadriceps muscle strength while providing analgesia similar to a conventional femoral nerve block for patients undergoing major knee surgery. Jaeger et al. showed that an adductor canal block produces a quadriceps strength reduction of 8% compared with 49% after a femoral nerve block [11].

In a randomized, double-blind study, the authors found that adductor canal block provides postoperative analgesia that is noninferior to femoral nerve block in the first 24 hours, while preserving quadriceps strength after outpatient ACL reconstruction [12].

## Conclusions

This study revealed that there is better pain control for ACL reconstruction in the immediate postoperative period with a combined femoral plus sciatic nerve block. However, further work is needed to include a larger possibly multicentered prospective randomized study, with a single anesthesiologist providing the blocks, and with surgery done at a single hospital and PACU staff. A study limited to a single anesthesiologist and single surgeon might help to decrease inevitable bias with three different practice patterns. Also, the future prospective studies should evaluate postoperative pain longer than during the PACU stay, a minimum of a week of daily follow-up and evidence of a rebound effect, quadriceps weakness, pain with movement, and with more standard times for pain assessment and treatment. To optimize pain management, adductor canal block (to decrease quadriceps weakness) and usage of a new formulation of local anesthetic, liposomal bupivacaine (now widely available and approved for wound infiltration), might be the future of regional anesthesia for postoperative pain in the pediatric population. Also, new prospective studies are needed with the new implemented federal and state regulations regarding fewer days of opioid prescriptions for acute pain management. Regional anesthesia can potentially help decrease the current opioid epidemic.
